# Antibacterial Activity and Molecular Docking of Lignans Isolated from *Artemisia cina* Against Multidrug-Resistant Bacteria

**DOI:** 10.3390/ph18060781

**Published:** 2025-05-23

**Authors:** Leslie Cynthia García Hernández, Rosa Isabel Higuera-Piedrahita, Nallely Rivero-Perez, Ana Lizet Morales-Ubaldo, Benjamín Valladares-Carranza, Héctor Alejandro de la Cruz-Cruz, Jorge Alfredo Cuéllar-Ordaz, Cynthia González-Ruiz, María Inés Nicolás-Vázquez, Adrian Zaragoza-Bastida

**Affiliations:** 1 Laboratorio 3 de la Unidad de Investigación Multidisciplinaria. Facultad de Estudios Superiores Cuautitlán, Universidad Nacional Autónoma de México, Carretera Cuautitlán-Teoloyucan km 2.5, San Sebastián Xhala, Cuautitlán 54714, Estado de México, Mexico; leslieghdz12@gmail.com (L.C.G.H.); delacruz@unam.mx (H.A.d.l.C.-C.); jcuellar@unam.mx (J.A.C.-O.); 2 Área Académica de Medicina Veterinaria y Zootecnia, Instituto de Ciencias Agropecuarias, Universidad Autónoma del Estado de Hidalgo, Rancho Universitario Av. Universidad km. 1, Ex Hacienda de Aquetzalpa, Tulancingo de Bravo 43660, Hidalgo, Mexico; nallely_rivero@uaeh.edu.mx (N.R.-P.); ubaldolizet8@gmail.com (A.L.M.-U.); 3 Facultad de Medicina Veterinaria y Zootecnia, Universidad Autónoma del Estado de México, El Cerrillo Piedras Blancas, Toluca 50090, Estado de Mexico, Mexico; bvalladaresc@uaemex.mx; 4 Laboratorio 8 de la Unidad de Investigación Multidisciplinaria. Facultad de Estudios Superiores Cuautitlán, Universidad Nacional Autónoma de México, Carretera Cuautitlán-Teoloyucan km 2.5, San Sebastián Xhala, Cuautitlán 54714, Estado de Mexico, Mexico; cgrmvz@hotmail.com; 5 Departamento de Ciencias Químicas, Facultad de Estudios Superiores Cuautitlán Campo 1, Universidad Nacional Autónoma de México, Av. 1o de Mayo s/n, Santa María las Torres, 54740, Estado de México, Mexico; nicovain@yahoo.com.mx

**Keywords:** Minimum Inhibitory Concentration, Minimum Bactericidal Concentration, 3′-demethoxy-6-O-demethylisoguaiacin, norisoguaiacin, molecular docking

## Abstract

The World Health Organization notes that some bacteria have been demonstrated to possess significant public health risks; they have antibiotic resistance, and there are fewer alternatives for control. The *n*-hexane extract and cinaguaiacin obtained from *Artemisia cina* show promising antibacterial activity, including against multidrug-resistant bacteria that affect animal and human health. **Objective:** The aim of this study was to determine the antibacterial activity of the *n*-hexane extract of *A. cina* and cinaguaiacin against multidrug-resistant bacteria. **Methods:** *A. cina* was collected in the pre-flowering period, the *n*-hexane extract was obtained, and chromatographic techniques and structure were used to separate the lignans, which were elucidated with nuclear magnetic resonance techniques. Four ATCC strains were used, and four strains were isolated from clinical cases with different resistance profiles. The antibacterial activity was determined by calculating the Minimum Inhibitory Concentration (MIC), Minimum Bactericidal Concentration (MBC), the time-kill kinetics assay, and the cell membrane integrity and DNA release assay. Molecular docking studies of lignans demonstrated the binding mode involved in the active site of DNA gyrase B. **Results:** The *n*-hexane extract inhibited growth against 87.5% of the strains tested (MIC 5.31 to 42.5 mg/mL) and showed bactericidal activity against 25% of the strains tested (MBC 0.62 to 85 mg/mL). Cinaguaiacin inhibited growth against 100% of the strains tested (MIC, 0.56 to 2.25 mg/mL) and exhibited bactericidal activity against 25% of the strains tested (MBC, 0.62 to 85 mg/mL). **Conclusions:** The mechanism of cinaguaiacin’s action may be associated with damage to the plasma membrane, as the protein and DNA levels were higher than those of the positive control. The n-hexane extract and cinaguaiacin obtained from *A. cina* showed a bacteriostatic or bactericidal effect, depending on the strain evaluated.

## 1. Introduction

The emergence of bacterial resistance to antibiotics has been a growing concern since the inception of the antibiotic era. The unregulated use of antibiotics has led to the frequent discovery of dangerous, antibiotic-resistant strains globally [1]. This has facilitated research on new potential antimicrobials, with medicinal plants being among the most promising sources [2]. Medicinal plants contain various chemical constituents, including alkaloids, flavonoids, terpenoids, and phenolic compounds, which exhibit diverse biological activities, including potent antibacterial properties [3].

Many different plants from diverse families have been studied, proving antibacterial effects against various bacteria, with *Staphylococcus aureus*, *Micrococcus flavus*, *Bacillus cereus*, *B. subtilis*, *Salmonella enteritidis*, *Escherichia coli*, and *Pseudomonas aeruginosa* being the most common ones [4,5,6]. Plants have long been a rich source of bioactive compounds, and among them, species of the genus *Artemisia* have garnered significant attention for their diverse pharmacological properties, including antibacterial activities [2,7,8].

The ethanolic extracts of the leaves and stems of *Artemisia absinthium* L. and *A. annua* L. have been proven to have antibacterial effects against *S. aureus*, *E. coli*, *Listeria monocytogenes*, and *S. enteritidis*, yielding promising results [9]. Moreover, the aqueous extracts of *A. monosperma*, *A. cina*, and *A. argyi* have also been reported to have activity against *E. coli*, *S. aureus*, *P. aeruginosa*, and *Enterococcus faecalis*, showing positive results for both Gram-positive and Gram-negative bacteria, with Gram-negative strains being the most susceptible [7].

*Artemisia cina* may represent a promising alternative treatment option due to its antibacterial properties; its ethyl acetate extract has demonstrated antibacterial effects against *E. coli*, *P. aeruginosa*, *B. subtilis*, and *S. aureus*, attributed to its flavonoid concentration [2]. Additionally, *A. cina* biosynthesizes other antibacterial compounds such as the lignan 3’-demethoxy-6-O-demethylisoguaiacin, identified in the *n*-hexane extract [10]. This lignan, previously reported in *Larrea tridentata*, exhibits antibacterial activity against resistant *S. aureus*, *L. monocytogenes*, *E. coli*, *P. aeruginosa*, *B. cereus*, *Klebsiella pneumoniae*, and *Pasteurella multocida* strains [8].

The compound described as 2,3-naphthalenediol, 5,6,7,8-tetrahydro-5-(4-hydroxyphenyl)-6,7-dimethyl-, [5R-(5α,6β,7β)]-, also known as (5R,6R,7R)-5,6,7,8-tetrahydro-5-(4-hydroxyphenyl)-6,7-dimethyl-2,3-naphthalenediol, and commonly referred to as 3′-demethoxy-6-O-demethylisoguaiacin, is found in the hexane extract of *Artemisia cina*. This compound was patented in 1989 under the CAS number 71113-16-1. Lignans have demonstrated significant activity as secondary metabolites produced by plants in response to stress, serving as a defense mechanism against fungi, insects, and even parasites.

The described lignan was reported again by Gnabre et al. [11] for its antiviral activity in humans. Later, Gnabre [12] patented the compound, providing evidence of its antiviral properties. Additionally, Garza-González et al. [13] reported antibacterial activity of the same compound, suggesting its potential as an alternative for controlling multidrug-resistant bacteria. Garza-González et al. [13] demonstrated that 3′-demethoxy-6-O-demethylisoguaiacin extracted from *Larrea tridentata* inhibits the propagation and growth of *S. aureus* (methicillin-resistant *S. aureus*), *Enterococcus faecalis*, *E coli*, *Enterobacter cloacae*, and isoniazid-resistant *Mycobacterium tuberculosis* [13]. This is particularly relevant given that antibacterial resistance is a global phenomenon affecting a large portion of the population.

Norisoguaiacin has a methoxy group at the 3’ carbon. Pardini et al. [14] demonstrated the effect of norisoguaiacin on inhibiting the electron transport system of mitochondria. This molecule, obtained from *Larrea divaricata*, was shown to inhibit the NADH oxidase enzyme in bovine heart mitochondria, as well as the succinoxidase enzyme; however, it did not inhibit cytochrome oxidase [14]. In 1974, Gisvold and Thaker reported the isolation of the lignans dihydroguaiaretic acid, norisoguaiacin, and 3′-demethoxyisoguaiacin from *Larrea divaricata* using sodium molybdate complexes. Furthermore, this lignan inhibits the enzymes formyltetrahydrofolate synthetase and carboxylesterase. The latter allows norisoguaiacin to inhibit phagocytosis and bind to several sites, preventing critical mitochondrial metabolic reactions [15].

*n*-Hexane extract and cinaguaiacina did not cause damage at therapeutic doses when administered orally to Wistar rats—the *n*-hexane extract of *A. cina* did not cause histopathological damage at pharmaceutical doses. However, the brain, kidneys, and liver are associated with biochemical parameters at higher doses (ten times the therapeutic dose). The lignans are proposed as antioxidant agents because they reduce the values in TBARS assays and increase the glutathione peroxidase values after oral administration.

In *A. cina*, 3′-demethoxy-6-O-demethylisoguaiacin is present in the *n*-hexane extract, mixed with norisoguaiacin (cinaguaiacin). Therefore, the present study aimed to determine the antibacterial activity of the *n*-hexane extract of *A. cina* and cinaguaiacin against multidrug-resistant bacteria.

## 2. Results

### 2.1. Extraction and Lignan Isolation of Artemisia cina

**Figure 1:**
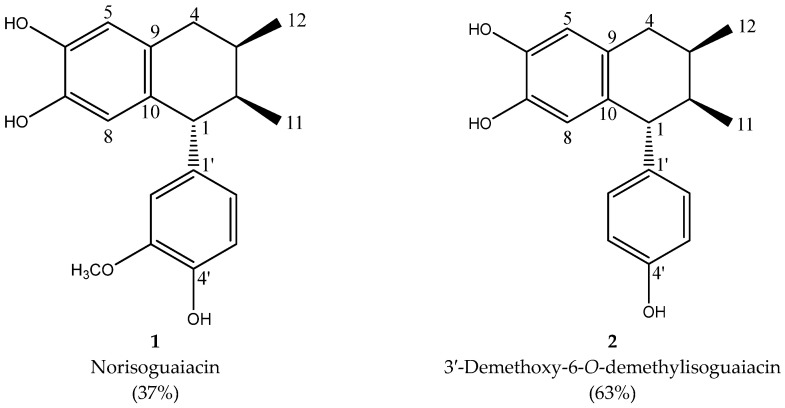
Structural design of the norisoguaiacin (compound **1**) and 3′-demethoxy-6-O-demethylisoguaiacin (compound **2**) mixture obtained from n-hexane extract of *Artemisia cina*.

The isolation of lignans was achieved by column chromatography; the mixture was composed of norisoguaiacin (compound **2**) and 3,3′-demethoxy-6-O-demethylisoguaiacin (compound **1**) at a ratio of 63:37, respectively. The structural design is shown in Figure 1.

**Figure 2:**
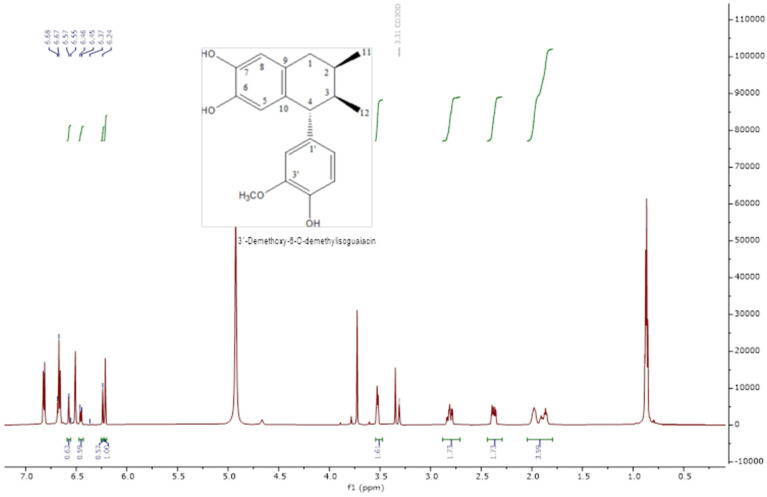
The 1H-NMR spectra of 2,3-naphthalenediol, 5,6,7,8-tetrahydro-5-(4—hydroxyphenyl)-6,7-dimethyl (isoguaiacin).

**Figure 3:**
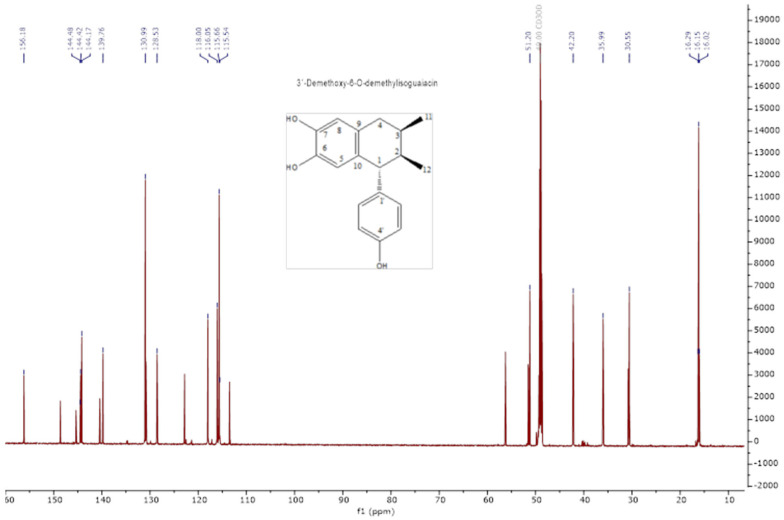
Carbon spectra (13C-NMR) of 2,3-naphthalenediol, 5,6,7,8-tetrahydro-5-(4—hydroxyphenyl)-6,7-dimethyl (isoguaiacin).

**Figure 4:**
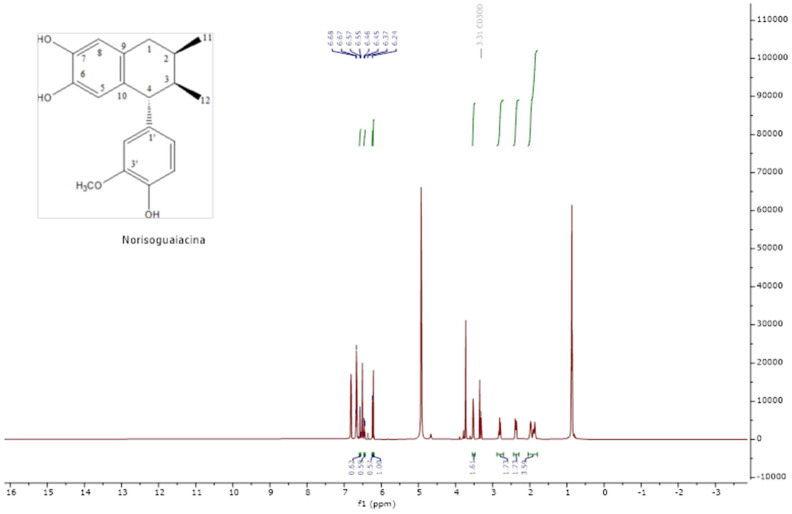
The 1H-NMR spectra of norisoguaiacin.

**Figure 5:**
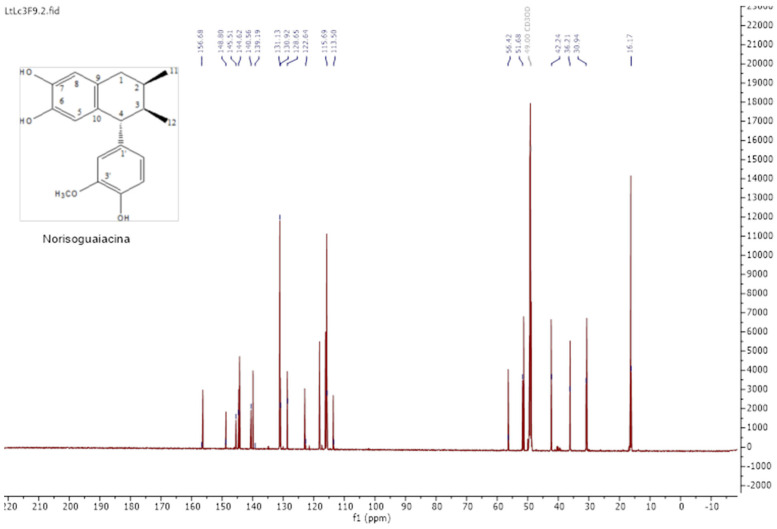
Carbon spectra (13C-NMR) of norisoguaiacin.

The analysis of the NMR spectra of 1H NMR and ^13^C NMR (Figure 2, Figure 3, Figure 4 and Figure 5) revealed the structures of 3′-demethoxy-6-O-demethylisoguaiacin (1) and norisoguaiacin (2). The experiments showed that compound **1** is present in the mixture at 63%, while the other compound is present at 37%. Table S1 shows the ^1^H-NMR (600 MHz) and ^13^C-NMR (150 MHz) spectral data of norisoguaiacin (1) and 3′-Demethoxy-6-O-demethylisoguaiacin (2) in CD_3_OD, along with the chemical structures of the molecules. The obtained spectra show high similarity to those reported by Higuera-Piedrahita et al. [10].

### 2.2. Antibacterial Activity

#### Minimum Inhibitory Concentration (MIC)

The MIC results showed statistically significant differences between the *n*-hexane extract and cinaguaiacin of *Artemisia cina*, as well as between their concentrations (*p* = 0.001). The *n*-hexane extract inhibited growth against 87.5% of the strains tested (7 of 8), in concentration ranges from 5.31 to 42.5 mg/mL, exhibiting the most significant inhibitory activity against *B. cereus* and the highest MIC against *S. Typhi*, *K. pneumoniae*, and *E. coli*^01^ (Table 1).

Cinaguaiacin inhibited growth against 100% of the strains tested, in concentration ranges from 0.56 to 2.25 mg/mL, exhibiting the most significant inhibitory activity against *B. cereus* and the highest Minimum Inhibitory Concentration (MIC) against *S. aureus*^02^ (Table 1).

### 2.3. Minimum Bactericidal Concentration (MBC)

The MBC results showed statistically significant differences between the *n*-hexane extract and cinaguaiacin of *Artemisia cina*, as well as between their concentrations (*p* = 0.001). The *n*-hexane extract exhibited bactericidal activity against 25% of the strains tested (2 of 8), in the concentration range of 0.62 to 85 mg/mL, with its best bactericidal activity against *B. cereus* and the highest Minimum Bactericidal Concentration (MBC) against *S. aureus*^02^ (Table 2).

Cinaguaiacin exhibited bactericidal activity against 50% of the strains tested (4 of 8), in the concentration range of 2.25 to 4.50 mg/mL, with its best bactericidal activity against *B. cereus* and the highest Minimum Bactericidal Concentration (MBC) against *S. aureus* strains (Table 2).

Regarding the bacteriostatic or bactericidal effect when relating to MBC/MIC, it was determined that the *n*-hexane extract presented a bactericidal effect; however, cinaguaiacin presented a bacteriostatic effect against 50% (4 of 8) of the strains and a bactericidal effect against 50% (4 of 8); the bactericidal effect was determined against the two multidrug-resistant strains of *S. aureus* and *B. cereus*.

### 2.4. Time-Kill Kinetics Assay

**Figure 6:**
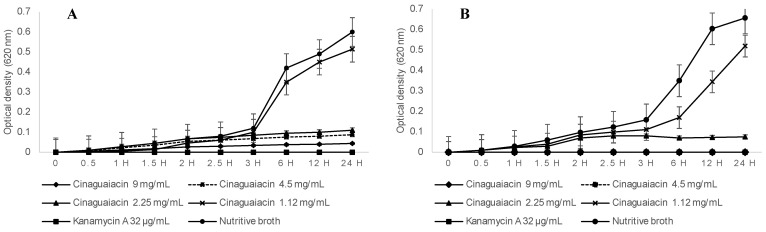
Time-kill assay of the cinaguaiacin on *E. coli* (**A**) and *S. aureus* (**B**).

Regarding the kill time for *E. coli*, statistically significant differences were determined between the cinaguaiacin concentrations and the controls. Similar growth was observed during the first 3 h; however, after this time, differences in growth became apparent. Cinaguaiacin at 1.12 mg/mL did not show statistical differences compared to the growth control during the monitoring period (*p* ≥ 0.05), as shown in Figure 6. No statistically significant differences were observed between the concentrations of 2.25, 4.5, and 9 mg/mL and the positive control (*p* ≥ 0.05). Growth remained constant, with no significant increase after 3 h.

Regarding *S. aureus*, statistically significant differences were determined between the cinaguaiacin concentrations and the controls. Concentrations of 1.12 and 2.25 mg/mL exhibited similar behavior to *E. coli* up to 2.5 h. After this time, exponential growth was observed for the 1.12 mg/mL concentration and the growth control, with no statistical differences at 24 h (*p* ≥ 0.05). The concentration of 2.25 mg/mL did not exhibit additional growth and remained constant throughout the evaluation period. Concentrations of 4.5 and 9 mg/mL did not show growth, coinciding with the positive control (*p* ≥ 0.05), as shown in Figure 6.

### 2.5. Cell Membrane Integrity and DNA Release

**Figure 7:**
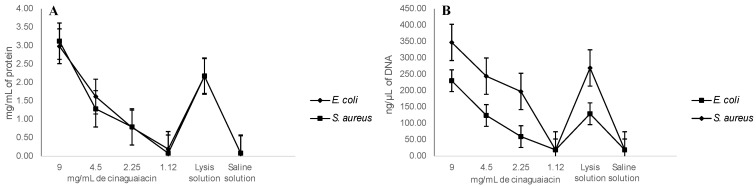
Leakage of proteins (**A**) and DNA (**B**) from *E. coli* and *S. aureus* treated with cinaguaiacin.

Regarding the membrane integrity, it was determined that, at 10 mg/mL of cinaguaiacin, 3.1 ± 0.46 mg/mL of protein was released for *E. coli*, and 2.99 ± 0.19 mg/mL was released for *S. aureus*—results that did not show significant statistical differences with the positive control (*p* ≥ 0.05). However, at concentrations of 5 and 2.5 mg/mL, smaller amounts of proteins were quantified, and even at 1.25 mg/mL no significant statistical differences were determined with the negative control (*p* ≥ 0.05) for both bacteria, as seen in Figure 7.

A greater release of DNA was determined at 10 mg/mL of cinaguaiacin: for *E. coli*, it was quantified at 347.5 ± 10 ng/µL, without showing statistical differences with the concentration of 5 mg/mL and the positive control (*p* ≥ 0.05); for *S. aureus*, 230.3 ± 10.7 ng/µL was quantified, a concentration higher than the positive control (*p* ≤ 0.05), and at 5 mg/mL 124.3 ± 9.6 µL was quantified, a concentration similar to that determined for the positive control (*p* ≥ 0.05) for this bacterium. For both bacteria, no significant statistical differences were observed at concentrations of 2.5 and 1.25 mg/mL for the negative control (*p* ≥ 0.05), as can be seen in Figure 7.

### 2.6. Molecular Docking

**Figure 8:**
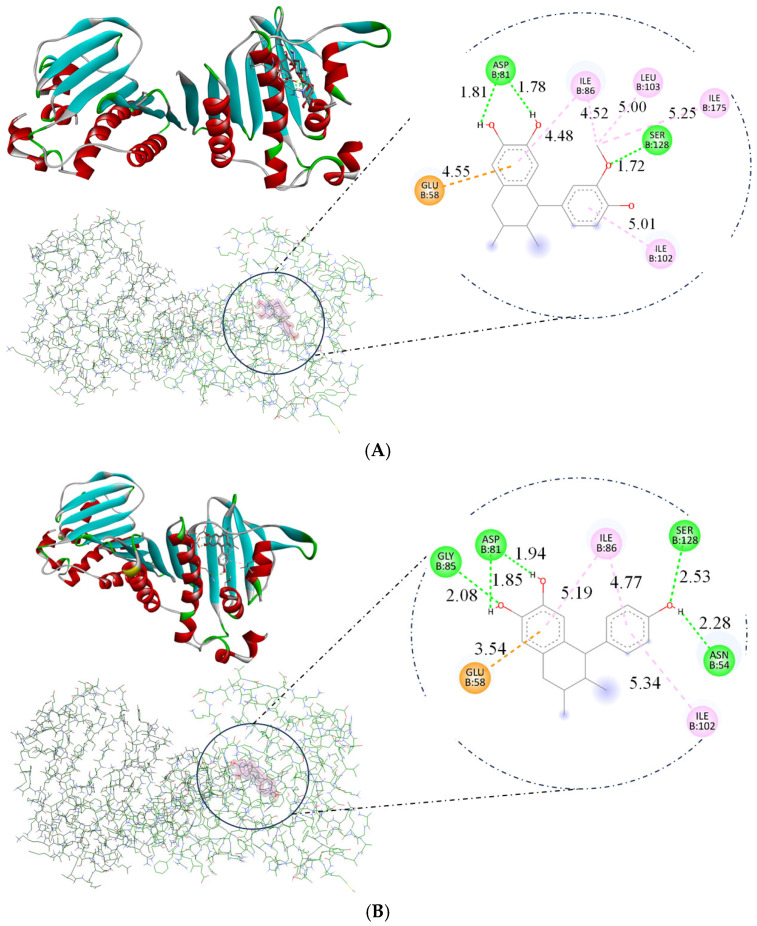
Binding modes of lignans obtained by docking studies with GyrB (PDB ID: 4URO): (**A**) Binding mode of norisoguaiacin (compound **1**) on the active site of GyrB, and amino acids of interaction. (**B**) Binding mode of 3′-demethoxy-6-O-demethylisoguaiacin (compound **2**) on the active site of GyrB, and amino acids of interaction.

The docking study directed in this research utilized DNA gyrase B with PDB ID 4URO to explore the mechanisms by which ligands act as antimicrobial agents. The compounds norisoguaiacin (1) and 3′-demethoxy-6-O-demethylisoguaiacin (2) exhibited similar affinity for DNA, as evidenced by their binding energy measurements, with a binding energy of −7.14 and −7.12 kcal/mol, respectively, which indicates interaction with the DNA structure, as illustrated in Figure 8. These interactions contribute to the compound’s antimicrobial activity, involving hydrogen bonds with critical amino acids such as Glu58, Asp81, and Gly85 [16,17]. In comparison, compounds **1** and **2** exhibited lower binding energies than kanamycin, as shown in Table 3. These differences in binding energy suggest that compounds **1** and **2** have weaker interactions with DNA; however, the experimental results indicate that compounds **1** and **2** can act as antimicrobial agents.

Molecular docking showed that compounds **1** and **2** can bind to GyrB, as shown in Figure 8. Hydrogen bonds were formed with ASP81 (bifurcated acceptor, 1.81 and 1.78 Å) and SER128 (1.72 Å) for compound **1**, and with ASN54 (2.28 Å), ASP81 (bifurcated acceptor. 1.85 and 1.94 Å), GLY85 (2.08 Å), and SER128 (2.53 Å) for compound **2**. The oxygen atoms of both lignans formed a hydrogen bond. Similar binding modes were also obtained for the GyrB inhibitor kanamycin. The GyrB part of compound **1** formed additional π-alkyl contacts with ILE86, ILE102, ILE175, and LEU103, while ILE86 and ILE102 formed contacts in compound **2**. Moreover, compounds **1** and **2** showed interaction with the anion at GLU58.

## 3. Discussion

Previous studies have demonstrated the potential antibacterial activity of different species of the *Artemisia* genus. For their part, in 2023, Bordean et al. [9] evaluated the antibacterial activity of *A*. *annua* and *A*. *absinthium* ethanolic extracts against *E. coli*, *S. aureus*, *L. monocytogenes*, and *S. enteritidis*. The authors found that *A. absinthium* showed inhibitory effects on *S. aureus* at a concentration of 25.0 mg/mL; a similar concentration was determined in our study against both reference and multidrug-resistant strains (MIC = 21.25 mg/mL), while better inhibitory effects were determined in the present study against *L. monocytogenes* (MIC = 21.25 mg/mL) in comparison with *A. absinthium* (MIC = 58.0–178.0 mg/mL). Concerning the Gram-negatives, the abovementioned study found activity at a range of inhibitory concentrations, from 54.0 to 375.0 mg/mL, higher than those reported in this study (42.50 mg/mL) [9].

A recent study assessed the effectiveness of *A. vestita* aqueous extract, determining its inhibitory effect at concentrations of 100, 150, and 200 µg/mL against *S. aureus*, *B. subtilis*, and *E. coli*, respectively. The results showed better activity against Gram-positive strains [18]. The *n*-hexane extract of *Artemisia afra* showed inhibitory activity against five different bacterial strains; the extract was active at concentrations from 0.156 to >2.50 mg/mL, and our hexane extract showed similar activity only against *B. cereus* when evaluated in this study [19].

For pure bioactive compounds, Morales-Ubaldo et al. [8] previously evaluated the activity of 3′-demethoxy-6-O-demethylisoguaiacin isolated from *L. tridentata*, which is generally considered to be more active than cinaguaiacin. Similarities between the two studies were identified; however, neither study determined antibacterial activity against the *S. aureus* ATCC strain. Nevertheless, the multidrug-resistant *S. aureus* strains were sensitive to the compound, albeit at different concentrations. Concerning *E. coli* (ATCC), cinaguaiacin was slightly more active, as evidenced by an MIC value of 1.12 mg/mL, which is lower than the previously reported value (MIC = 1.56 mg/mL). On the other hand, the observed differences in the activity of the compound may, therefore, be attributable to the differences in extraction procedures and the vegetal source [8].

As can be seen, and according to the literature to date, there are no recent reports regarding the activity of *A. cina* extracts and its related compounds, and even less so about combinations thereof. Nevertheless, it is well known that combinations of two or more phytoactives can bring about changes in the biological effects. In most cases, said mixtures provide an increase in the activity of interest [20].

In the present study, the combination named cinaguaiacin resulted in improved antibacterial activity. The mixture was 76 times more active against *L. monocytogenes* than the *n*-hexane extract, and its effectiveness against *S. typhi*, *K. pneumoniae*, and *E. coli* also increased (38 times). Against *S. aureus*, the mixture was up to 19 times more active, and it was 10 times more active against *B. cereus*. Combinatory therapies enhance the antibacterial spectrum and increase the bioavailability of antibacterial agents within bacterial cells, leading to the successful control of multidrug-resistant strains [21].

Additionally, to determine the antibacterial effects of cinaguaiacin, the mode of action was determined through time-kill kinetics, cell membrane integrity, and DNA release assays, providing a better viewpoint on the potential bactericidal activity of cinaguaiacin. In the present study, a concentration-dependent response was determined for both bacteria, indicating that the activity is significant and adjustable according to the dosage, and reasserting the expectation that a more concentrated sample will kill bacteria in a shorter period. This may be associated with the obtained data regarding cell membrane integrity. Some authors have stated that an increase in the concentrations of phytochemicals leads to an increase in the diffusion of the bioactives into the cell membrane, causing membrane destruction. In previous studies, it was demonstrated that *Artemisia*-based treatments can damage the cytoplasmic membrane in bacteria, causing loss of vital chemicals and cell death [22,23,24].

Favela-Hernández et al. [25] demonstrated that the lignan isolated in their study exhibited a mechanism of action targeting *S. aureus*. Using microarray methodology, they showed that the lignan’s activity was localized in the bacterial cell membrane, specifically affecting ATP-binding transport proteins, also known as ABC transporters. This disruption prevents the bacteria from utilizing ATP, ultimately leading to their death. This mechanism is novel, as no existing antibacterial agent operates in this manner, making it a promising candidate for new therapeutic approaches [25].

In addition to the benefits of 3’-demethoxy-6-O-demethylisoguaiacin, Luna-Vázquez et al. [26] demonstrated that the compound induces the relaxation of vascular smooth muscle, suggesting its potential as an antihypertensive drug. They proposed that its mechanism of action involves the nitric oxide (NO) pathway and the expression of secondary messengers such as cyclic guanosine monophosphate (cGMP), along with the modulation of H2S- and ATP-sensitive potassium channels in the physiological vasodilation cascade.

Moreover, they identified an endothelium-independent vasodilation pathway, which remains to be fully elucidated and may represent a secondary mechanism of action. Gnabre et al. [11] observed that norisoguaiacin possesses antiviral properties, specifically against HIV, a disease of significant concern to humans. Similarly, Torres et al. [27] demonstrated that lignans extracted from *Larrea nitida*, such as norisoguaiacin and meso-nordihydroguaiaretic acid, exhibit antioxidant activity when used as resins or in their pure form. Their study focused on ABTS transporters, which generate cationic radicals [27].

Schmidt et al. [28] found that the described lignan has anti-protozoal properties. They used a dichloromethane extract from *Larrea tridentata* to test against *Trypanosoma brucei rhodesiense*, *Trypanosoma cruzi*, *Leishmania donovani*, and *Plasmodium falciparum*. While the lignan showed cytotoxic effects on rat myoblasts at a dose of 25.4 µg/mL, its therapeutic doses (LD_50_) were 2.8, 14.6, 5.2, and 2.9 µg/mL, respectively, for each parasite. The most abundant and active lignan in *L. tridentata* was identified as meso-nordihydroguaiaretic acid, which is also attributed with anti-inflammatory properties [28].

*Artemisia* plants have been used to treat bacterial infections in humans since ancient times; however, only artemisinin from *A. annua* has been widely recognized for its antimicrobial efficacy [24]. In this context, the present study offers essential insights into the bactericidal effects of cinaguaiacin. It is imperative to emphasize the need for future research to elucidate therapeutic applications of cinaguaiacin.

Docking studies enabled the distinction of the distinct modes of interaction exhibited by these derivatives with the amino acid backbone in the DNA-binding site, highlighting the importance of oxygen atoms in facilitating molecular binding to DNA gyrase. This suggests that the presence of such amino acids may enhance the efficacy of the compounds in targeting bacterial DNA. The ΔG (G) of kanamycin, as determined in this docking study, showed a higher yield due to nine hydrogen bond interactions (1.87–3.05 Å). Lignans showed fewer hydrogen bonds—three for one and five for the other—but these compounds exhibit other interactions that could enhance their antimicrobial activity.

## 4. Materials and Methods

### 4.1. Plant Material

The fresh pre-flowering leaves and stems of *A. cina* O. Berg ex Poljakov (Asteraceae) were sourced from the Hunab laboratory, totaling 10 kg. A voucher specimen, authenticated by Dr. Alejandro Torres-Montúfar, has been deposited at the herbarium of the Facultad de Estudios Superiores Cuautitlán (FES-C) UNAM, México, under voucher no. 11967. The plant was cultivated under conditions of 80% humidity, a temperature of 24 °C, and soil with a pH of 6.3.

### 4.2. Extraction and Lignan Isolation of Artemisia cina

The dried leaves and stems of *A. cina* O. Berg ex Poljakov (Asteraceae) were macerated with *n*-hexane for 24 h. The plant was cultivated under conditions of 80% humidity, a temperature of 24 °C, and soil with a pH of 6.3. Chromatographic techniques were used to separate the dry extract, using n-hexane and ethyl acetate in a descending mode. The fractions obtained were concentrated and evaluated by thin-layer chromatography. The fraction identified as containing lignans was concentrated and lyophilized to eliminate the solvent. The fraction was refrigerated and elucidated by nuclear magnetic resonance. The chromatograms were interpreted, and the Mest-Renova^®^ program drew the structures of these molecules.

### 4.3. Bacterial Strains

As biological material, four ATCC strains were used *(Escherichia coli*^35218^, *Staphylococcus aureus*^6538^, *Listeria monocytogenes*^19113^, and *Salmonella enterica* serovar Typhimurium^14028^), along with four multidrug-resistant strains isolated from ovine clinical cases (*Bacillus cereus*, *Klebsiella pneumoniae*, *Escherichia coli*^01^, and *Staphylococcus aureus*^02^) from the collection of the Bacteriology Laboratory of the Academic Area of Veterinary Medicine and Zootechnics at the Autonomous University of the State of Hidalgo. These strains were isolated from clinical cases with reports of antimicrobial resistance, according to Morales-Ubaldo et al. [8]. The strains were cryopreserved at −80 °C until use.

### 4.4. Antibacterial Activity

The antibacterial activity was determined by calculating the Minimum Inhibitory Concentration (MIC), Minimum Bactericidal Concentration (MBC), and the MBC/MIC ratio, following the guidelines established by the CLSI (2012), and as reported by Zaragoza-Bastida et al. [29,30].

#### 4.4.1. Sterility Test

The sterility of each treatment was assessed by inoculating 10 µL of each sample onto Mueller–Hinton agar (BD Biosciences, Heidelberg, Germany), followed by incubation at 37 °C for 24 h to detect any microorganisms present in the sample. In the case of microbial growth in the inoculation zone, the sample underwent sterilization using two membrane filters: one with a 33 mm diameter, and the other with a 0.22 µm pore size (Millex-GV).

#### 4.4.2. Reactivation of Bacterial Strains

Each bacterial strain was reactivated from cryopreservation on Mueller–Hinton agar (BD Bioxon, Heidelberg, Germany) using the simple streak technique to obtain isolated colonies. They were then incubated for 24 h at 37 °C.

#### 4.4.3. Preparation of Inoculum

Once the purity of each bacterium was confirmed, a single colony of each strain was inoculated into the nutrient broth (BD Bioxon, Heidelberg, Germany), which was then incubated with constant agitation (70 rpm) for 24 h at 37 °C. After the incubation period, the inoculum was adjusted with nutrient broth to a 0.5 McFarland turbidity standard (Remel, R20421, Lenexa, KS, USA), corresponding to 1.5 × 10^6^ cells/mL.

#### 4.4.4. Minimum Inhibitory Concentration (MIC)

The microdilution method was used in a plate to determine the MIC. For the hydroalcoholic extract, the concentrations ranged from 0.62 to 170 mg/mL, while for the hexane extract and cinaguaiacin, the concentrations ranged from 9 to 0.28 mg/mL. Sterile nutrient broth served as the negative control, and kanamycin A sulfate salt (AppliChem 4K10421, Darmstadt, Germany) was used as the positive control.

Each treatment was evaluated in triplicate in a 96-well plate. For this, 100 μL of each concentration to be tested, plus 10 μL of the bacterial suspension adjusted to 0.5 McFarland, was added to each well. Once inoculated, the plate was incubated at 37 °C for 24 h with constant agitation (70 rpm).

To determine the MIC endpoint, a colorimetric method using p-iodonitrotetrazolium salts (Sigma-Aldrich 18377 (St. Louis, MO, USA), EEUU) was employed. After the incubation period, 20 μL of a 0.04% (*w*/*v*) solution of p-iodonitrotetrazolium was added to each well, and the plate was further incubated for 30 min at the corresponding temperature for each bacterium. The MIC was determined as the concentration at which the solution turned a pink color. Tetrazolium salts were used as colorimetric indicators, as bacteria or fungi convert them into colored formazan derivatives through redox reactions.

#### 4.4.5. Minimum Bactericidal Concentration (MBC)

To determine the MBC, after the addition of p-iodonitrotetrazolium, 5 μL from each well was inoculated onto Mueller–Hinton agar (Difco (Beyrouth, Lebanon), EEUU). The plates were then incubated at 37 °C for 24 h. After the incubation period, readings were taken, and the MBC was determined as the concentration at which no bacterial growth was observed on the plate. The MBC is defined as the lowest concentration of an antimicrobial agent required to kill 99.9% of the inoculum, with no visible bacterial growth on the plate after 24 h [31].

### 4.5. Time-Kill Kinetics Assay

Cinaguaiacin was the most active treatment; therefore, it was determined that the time-kill assay used *E. coli*^35218^ and *S. aureus*^6538^ suspensions as indicator bacteria, as described in [32]. The final concentrations were 1.12, 2.25, 4.5, and 9 mg/mL. Kanamycin A at 32 µg/mL was used as a positive control, and nutritive broth with bacteria was used as a negative control for growth. Into a 96-well plate, 100 µL of each concentration was added, followed by the addition of 10 µL of the bacterial suspension, adjusted to a 0.5 McFarland standard. The plates were incubated at 37 °C, the measurements were at 0, 0.5, 1, 1.5, 2, 2.5, 3, 6, 12, and 24 h, and the optical density at 620 nm was measured [32].

### 4.6. Cell Membrane Integrity and DNA Release

Damage to bacterial cell membrane integrity by cinaguaiacin was examined by measuring the release of proteins and DNA, indicating leakage through the bacterial membrane. Bacterial cells in the logarithmic growth phase (*E. coli*^35218^, *S. aureus*^6538^) were subjected to treatment with the cinaguaiacin at 1.12, 2.25, 4.5, and 9 mg/mL. Cell lysis solution (Promega, Madison, WI, USA) was used as a positive control, and saline solution (0.9%) was used as a negative control.

The treatments had an incubation period of 24 h at a temperature of 37 °C. Following the incubation, the bacteria were separated from the supernatant by centrifugation at 10,000× *g* for 5 min. The concentrations of proteins and DNA in the supernatant were quantified using a NanoDrop Thermo Fisher Scientific 1000 (Wilmington, DE, USA) with an absorbance of 280 nm and 260 nm, respectively [32].

### 4.7. Statistical Analysis

The results obtained from the antibacterial activity were normalized (log10) and analyzed using a completely randomized design via analysis of variance (ANOVA). Differences between means were assessed using Tukey’s statistical comparison with a significance level of 95% (*p* ≤ 0.05), as determined by SAS version 9.0 software.

### 4.8. Quantum Chemical Calculations

The lignan structures were modeled using Spartan’06 (Wavefunction Inc., Irvine, CA, USA, 2006). Computational chemistry calculations were also conducted within this software, employing semi-empirical methods such as the AM1 (Austin Model 1) conformational analysis to determine molecular stability. As a result, a set of local minimum potential energy conformers was generated in the gas phase. After analysis, the lowest-energy conformers (in kcal/mol) were selected for further optimization.

Using GaussView06 (Dennington II, Keith, and Millam, 2016) the molecular coordinates (x, y, z format) of the previously selected conformers for each lignan were obtained. These coordinates were sent to the Miztli supercomputer at the Universidad Nacional Autónoma de México (UNAM, Mexico City, Mexico, n.d.). The lignan structures and their energies were calculated using the hybrid density functional model (DFT/B3LYP) and the extended basis set 6-311++G(d,p). This is a triple-valence basis set augmented with d-polarization functions on heavy atoms such as carbon (C) and oxygen (O), p-polarization functions (parallel polarization) on hydrogen (H) atoms, and diffuse functions applied to all atoms.

### 4.9. DNA Gyrase B Preparation

The three-dimensional structure of DNA gyrase B (PDB ID: 4URO) was obtained from the Protein Data Bank database (Research Collaboratory for Structural Bioinformatics (CSB, n.d.). Discovery Studio 2019 (Dassault Systèmes BIOVIA, 2019) and other online software tools were used to remove water molecules and unwanted ligands. Moreover, since the B subunit of DNA gyrase (GyrB) forms a functional homodimer, meaning that it is a structure formed by the binding of two identical subunits, these two subunits were divided into four protein chains, in this case (as shown in Discovery Studio) labeled A, B, C, and D, where A and B are identical, while C and D are also similar to each other. Therefore, only one subunit was worked with to facilitate the docking calculations. Parts A and D were removed, retaining the protein chains B and C.

### 4.10. Docking Studies

The docking study determined the interactions of our lignan molecules with the DNA gyrase B protein. Molecular docking studies represent an advanced computational tool used to model and predict, at an atomic level, how a molecule (ligand) interacts with a target protein. This approach is essential in drug design, as it enables the exploration of the optimal conformation of the protein–ligand complex and the estimation of binding affinity. Additionally, these studies help identify potential active sites in the protein and assess the efficacy of candidate compounds.

For the docking studies, the AutoDock Tools software (Vina v1.2.x (2021–present) was used. This tool operates by keeping the macromolecule (typically a protein) in a rigid conformation while allowing the ligand to maintain flexibility, adjusting its rotations and torsions to explore various spatial configurations. This ligand’s flexibility is crucial for realistically simulating the possible interactions that would occur in a biological environment. A grid-map-based approach was used to generate the necessary structural entries and accurately delineate all possible interaction sites within the protein, utilizing a rectangular grid for each of the selected protein chains (B and C). For B (108 × 124 × 115 Å) and C (122 × 105 × 122 Å), both were separated by 0.375 Å, focusing on the active site of DNA gyrase B.

## 5. Conclusions

The *n*-hexane extract and cinaguaiacin obtained from *A. cina* showed a bacteriostatic or bactericidal effect, depending on the strain evaluated. It was determined that the n-hexane extract had a bacteriostatic effect against 100% of the strains evaluated and a bactericidal effect against only 15%. However, cinaguaiacin had a bacteriostatic effect against 100% of the strains and a bactericidal effect against 50% of them, including multidrug-resistant strains. The cinaguaiacin had a Minimum Bactericidal Concentration of 1.12 mg/mL in *B. cereus* strains, and in ATCC reference strains it was 2.25 mg/mL for *S. typhi*. Compounds **1** and **2** underwent molecular docking studies, which revealed binding to the active site of DNA gyrase. However, additional studies are required to verify this mechanism of action and its cytotoxic effect before it can be evaluated in an in vivo model.

## Figures and Tables

**Table 1:** The Minimum Inhibitory Concentration of *Artemisia cina n*-hexane extract and cinaguaiacin.

	Bacteria	*n*-Hexane Extract (mg/mL)	Cinaguaiacin (mg/mL)	Kanamycin A (µg/mL)
**Reference ATCC**	*E. coli*	NA	1.12 ^bC^	1^aB^
	*S. typhi*	42.50 ^cC^	1.12 ^bC^	2 ^aC^
	*S. aureus*	21.25 ^cB^	1.12 ^bC^	2 ^aC^
	*L. monocytogenes*	21.25 ^cB^	0.28 ^bA^	0.5 ^aA^
**Multidrug-resistant**	*B. cereus*	5.31 ^cA^	0.56 ^bB^	0.5 ^aA^
	*K. pneumoniae*	42.50 ^cC^	1.12 ^bC^	2 ^aC^
	*E. coli* ^01^	42.50 ^cC^	1.12 ^bC^	2 ^aC^
	*S. aureus* ^02^	21.25 ^cB^	2.25 ^bC^	4 ^aD^
	*p*-Value	0.001	0.001	0.001

NA = no activity; ^a,b,c^ different letters in the rows indicate significant statistical differences (*p* ≤ 0.05); ^A,B,C^ different letters in the columns indicate significant statistical differences (*p* ≤ 0.05).

**Table 2:** The Minimum Bactericidal Concentration of *Artemisia cina n*-hexane extract and cinaguaiacin.

	Bacteria	*n*-Hexane Extract (mg/mL)	Cinaguaiacin (mg/mL)	Kanamicin A (µg/mL)
**Reference ATCC**	*E. coli*	NA	NA	2 ^aB^
	*S. Typhi*	NA	2.25^bB^	4 ^aC^
	*S. aureus*	NA	4.50 ^bC^	2 ^aB^
	*L. monocytogenes*	NA	NA	4 ^aC^
**Multidrug-resistant**	*B. cereus*	0.62 ^bA^	1.12 ^CA^	1 ^aA^
	*K. pneumoniae*	NA	NA	4 ^aC^
	*E. coli* ^01^	NA	NA	4 ^aC^
	*S. aureus* ^02^	85.0^cB^	4.50 ^bC^	8 ^aD^
	*p*-Value	0.001	0.001	0.001

NA = no activity; ^a,b,c^ different letters in the rows indicate significant statistical differences (*p* ≤ 0.05); ^A,B,C^ different letters in the columns indicate significant statistical differences (*p* ≤ 0.05).

**Table 3:** Amino acids’ ΔG values obtained by docking studies between lignans and GyrB.

Compound	ΔG (kcal/mol)	Amino Acids Interact with GyrB
**Kanamycin**	−9.6	ASN54, ASP57, GLU58, ASP81, SER128
**1**	−7.14	GLU58, ASP81, ILE86, ILE102, LEU103, SER128, ILE175
**2**	−7.12	ASN54, GLU58, ASP81, GLY85, ILE86, ILE102, SER128

## Data Availability

The databases used and/or analyzed during the current study are available from the corresponding author upon reasonable request. The data is not public since it is under intellectual property protection.

## Supplementary Materials

The following supporting information can be downloaded at: https://www.mdpi.com/article/10.3390/ph18060781/s1, Table S1. Spectral data of ^1^H-NMR (600 MHz) and ^13^C-NMR (150 MHz) 3′-Demethoxy-6-O-demethylisoguaiacin (**1**) and norisoguaiacin (**2**) in CD_3_OD and chemical structures of molecules.
