# Supplementary material for: Antibacterial Activity and Molecular Docking of Lignans Isolated from Artemisia cina Against Multidrug-Resistant Bacteria
Source: Pharmaceuticals (Basel). 2025 May 23;18(6):781. doi: 10.3390/ph18060781 (PMC12196185; doi:10.3390/ph18060781)
Supplement: Supplementary file 1 [file pharmaceuticals-18-00781-s001.zip › pharmaceuticals-3593176-supplementary.pdf]

Powerful Antibacterial Lignans from *Artemisia cina* Against Multidrug-Resistant Bacteria

Leslie Cynthia García Hernández[1], Rosa Isabel Higuera-Piedrahita[1*], Nallely Rivero Pérez[2], Ana Lizet Mora-les-Ubaldo[2], Benjamín Valladares-Carranza[3], Héctor Alejandro de la Cruz-Cruz[1], Jorge Alfredo Cuéllar-Ordaz[1], Cynthia González-Ruiz[1], María Inés Nicolás-Vázquez[2], and Adrián Zaragoza-Bastida[2*]

[1] Laboratorio 3 de la Unidad de Investigación Multidisciplinaria. Facultad de Estudios Superiores Cuautitlán, Universidad Nacional Autónoma de México, Carretera Cuautitlán-Teoloyucan km 2.5, San Sebastián Xhala, Cuautitlán 54714, Estado de México, México.

[2]Área Académica de Medicina Veterinaria y Zootecnia, Instituto de Ciencias Agropecuarias, Universidad Autónoma del Estado de Hidalgo. Rancho Universitario Av. Universidad km. 1, Ex Hacienda de Aquetzalpa, Tulancingo de Bravo 43660, Hidalgo, México.

[3]Facultad de Medicina Veterinaria y Zootecnia, Universidad Autónoma del Estado de México, El Cerrillo Piedras Blancas, Toluca 50090, Estado de Mexico, Mexico.

[4]Departamento de Ciencias Químicas, Facultad de Estudios Superiores Cuautitlán Campo 1, Universidad Nacional Autónoma de México, Av 1o de Mayo s/n, Santa María las Torres, Cuautitlán Izcalli, Estado de México, 54740, México.

Table S1. Spectral data of ^{1}H-NMR (600 MHz) and ^{13}C-NMR (150 MHz) 3'- Demethoxy-6-O-demethylisoguaiacin (**1**) and norisoguaiacin (**2**) in CD_3OD and chemical structures of molecules.

Position	**1** δ_H (J in Hz) 600 MHz	**1** δ_C 150	**2** δ_H (J in Hz) 600 MHz	**2** δ_C 150
1	3.53 (1H, d, 6.2)	51.3	3.53 (1H, d, 5.1)	51.6
2	1.86, s	42.3	1.90 (m)	42.2
3	1.98 (m)	30.7	1.98 (m)	30.9
4 a<br>B	2.8 (1H, dd, 5.1, 16.1)<br>2.3 (1H, dd, 6.6, 16.1)	36.1	2.82 (1H, dd, 5.1, 12.8)<br>2.38 (1H, dd, 4.7, 16.1)	36.2
5	6.51, s	116.2	6.51 (1H, s)	118.1
6		144.5		144.6
7		144.3		144.3
8	6.21, s	118.1	6.34, (1H, s)	116.2
9		130.9		130.9
10		128.6		128.6
11	0.86 (3H, d, 6.2)	16.3	0.88 (3H, d, 5.1)	16.1
12	0.87 (3H, d, 6.2)	16.3	0.86 (3H, d, 6.2)	16.4
1’		139.9		140.6
2’	6.82 (1H, d, 8.4)	131.14	6.57 (1H, br, s)	113.6
3’	6.67 (1H, d, 8.4)	115.8		148.7
4’		156.3		145.5
5’	6.67 (1H, d, 8.4)	115.8	6.68 (1H, d, 7.7)	115.7
6’	6.82 (1H, d, 8.4)	131.14	6.45 (1H, d, br, 7.7)	122.9
OCH_3			3.7 (3H, s)	56.4
